# Psychopathy dimensions and substance use: evidence from a non-institutionalized adult population

**DOI:** 10.3389/fpsyt.2025.1670589

**Published:** 2025-12-11

**Authors:** Yara El Frenn, Roua Abbas, Zuhair Hatahet, Khalil El Asmar, Elias Ghossoub

**Affiliations:** 1 Department of Psychiatry, Faculty of Medicine, American University of Beirut, Beirut, Lebanon; 2 Faculty of Medicine, American University of Beirut, Beirut, Lebanon; 3 Department of Epidemiology and Biostatistics, American University of Beirut, Beirut, Lebanon; 4 Le Centre de Recherche en Epidémiologie et Santé des Populations (CESP), MOODS Team, INSERM UMR 1018, Faculté de Médecine, Univ. Paris-Saclay, Le Kremlin Bicêtre, France

**Keywords:** psychopathy, substance abuse, Lebanon, angsocial factor, callousness

## Abstract

**Introduction:**

Mental health in Lebanon has been profoundly affected by recent crises, altering the patterns of substance use and psychiatric presentations. In this context, the link between psychopathy and substance use is relevant, but is underexplored in Arab cultural settings. This study aimed to assess the relationship between psychopathy and substance use in the Arab world, focusing on whether individuals in a Lebanese non-institutionalized population who use substances have higher psychopathy traits than those who do not.

**Methods:**

This is a cross-sectional study conducted among 534 non-institutionalized Lebanese adults between May and June 2021. Participants completed a self-administered questionnaire including the validated Levenson Self-Report Psychopathy (LSRP) scale, TAPS (the Tobacco, Alcohol, Prescription medication, and Other substance use screener), the short version of the Urgency, Premeditation (lack of), Perseverance (lack of), Sensation Seeking, Positive Urgency, Impulsive Behavior scale (S-UPPS-P), and the Adverse Childhood Experiences (ACE) questionnaire. Bivariate analysis and multivariable regression were used to assess the association between psychopathy traits and substance use, adjusting for relevant factors.

**Results:**

The mean age of the participants was 36.58 ± 11.94 years, and approximately 80% were women. Callousness was linked with tobacco, alcohol, and illicit drug use, while antisocial traits were associated with tobacco and prescription drug misuse. The regression analyses revealed that the antisocial factor was robustly associated with overall substance use (OR = 1.07, 95% CI = 1.01–1.14, p = 0.02), including tobacco use (OR = 1.13, 95% CI = 1.05–1.21, p = 0.001) and prescription drug misuse (OR = 1.28, 95% CI = 1.14–1.43, p < 0.001). Moreover, it showed that callousness significantly increased the odds of alcohol (OR = 1.15, 95% CI = 1.05–1.26, p = 0.003) and illicit drug use (OR = 1.13, 95% CI = 1.03–1.24, p = 0.01).

**Discussion:**

This is the first study that examined the relationship between substance use and psychopathy traits in a Lebanese population. These results argue for trait-specific interventions to improve the efficacy and cost-effectiveness of substance use prevention and rehabilitation programs.

## 1 Introduction

Lebanon’s mental health landscape has undergone dramatic changes in recent years, shaped by multiple crises including economic collapse, political instability, and the coronavirus disease 2019 (COVID-19) pandemic. These challenges have significantly impacted the patterns of substance use and mental health presentation, creating a unique context for understanding personality disorders and their relationship with substance use. Similar patterns observed elsewhere showed that major collective traumas lead to measurable shifts in psychiatric admissions and diagnostic profiles, including increased presentations related to stress, mood, and substance use (1). It is within this complex environment that this study seeks to examine the relationship between psychopathy and substance use, a link well documented in the West, but is largely unexplored in Arab cultures.

For this purpose, we employed the dominant Western model of psychopathy, which defines the construct through three core features: interpersonal, affective, and behavioral (2). Interpersonal characteristics include manipulation and deceitfulness (3), while affective traits encompass callousness and lack of empathy or guilt (4). The behavioral dimension presents as impulsivity and law-breaking tendencies (2). Understanding how these traits manifest and interact with substance use in non-Western cultures is crucial for the development of culturally appropriate interventions.

Substance use disorders, which are characterized by harmful patterns of use that impair physical and mental health (5), present unique challenges in the Lebanese context. While these disorders are prevalent worldwide with varying rates across countries (6), their manifestation in Lebanon reflects distinct cultural, social, and economic factors. Traditional gender patterns of substance use in the Arab world, where higher rates are typically observed in men, may be shifting due to recent societal changes (7). Although nicotine and alcohol use disorders remain prevalent globally (8), the patterns and types of substance use in Lebanon may differ due to cultural norms, accessibility, and economic factors.

Psychopathy often coexists with substance use disorders (9), and understanding this association is crucial for improving treatment outcomes. While Western research has established that individuals with psychopathy often begin substance use early and tend toward polysubstance dependence (10–13), the generalizability of these findings to Arab populations remains uncertain. Studies in Western contexts have found significantly higher rates of psychopathy (5%–40%) among individuals with substance use disorders compared with only 1% in the general population (9). However, in Lebanon, these patterns may manifest differently due to unique cultural factors, healthcare system characteristics, and current societal challenges.

Multiple tools have been designed for the assessment of psychopathy, with the Psychopathy Checklist—Revised (PCL-R) being the gold standard (14, 15). A more novel tool, the Levenson Self-Report Psychopathy (LSRP) scale, describes a dual-factor model of psychopathy, with the primary factor encompassing interpersonal and affective features and the secondary factor including antisocial and lifestyle features (16). Our team validated the LSRP in a sample of a non-institutionalized Lebanese population (17). The results yielded a three-factor (egocentric, callous, and antisocial), 25-item model in comparison to the original 26-item scale (17).

A recent review on the epidemiology of psychopathy and antisocial personality disorder in the Arab region has highlighted the scarcity of research in the field (18). To our knowledge, this study is the first to evaluate the association between psychopathy and substance use in the Arab world. Our main objective was to assess whether individuals in a Lebanese non-institutionalized population who use substances have higher psychopathy traits than those who do not.

## 2 Methods

### 2.1 Aim, design, and setting of the study

This cross-sectional study aimed to assess whether individuals in a Lebanese non-institutionalized population who use substances have higher psychopathy traits than those who do not. Participants were recruited via online advertising on social media platforms including Facebook, X (formerly Twitter), and Instagram.

### 2.2 Participants

A total of 1,541 individuals clicked on the link to access the survey, of which 572 participants filled out the survey. Included in this study are Lebanese adults residing in Lebanon, aged 18–65 years, representing the population on which the LSRP was validated by the initial authors.

Those who are not Lebanese and Lebanese nationals living outside of Lebanon were excluded. A total of 534 Lebanese participants completed at least 80% of the items in all the scales, and these were included.

### 2.3 Procedure

This study is a secondary analysis of the dataset used for “Validation of the Levenson Self-Report Psychopathy (LSRP) scale in the non-institutionalized Lebanese population” (17). The Institutional Review Board (IRB) of the American University of Beirut approved both the primary study (ID: SBS-2020-0491) and the secondary study (ID: SBS-2024-0216). In our survey, anonymous participation was ensured due to the sensitive nature of the information related to participants’ past experiences, and no private health information was collected. Data collection extended from May to June 2021. The data are stored on the principal investigator’s password-protected laptop and will be kept up to 3 years after study completion, after which these will be discarded. Convenience sampling was used to invite participants to complete an online self-administered questionnaire through the online LimeSurvey platform. The LimeSurvey platform allows one submission per participant to prevent multiple responses. Participants were informed about the study’s objectives and inclusion criteria, after which interested individuals read and agreed to an online consent form.

### 2.4 Measures

All scales were available in both English and Arabic. The back-translation method was used for accuracy and cultural relevance of the Arabic scales. Two bilingual translators completed the forward translation, and two others performed the backtranslation. Discrepancies were resolved through consensus discussions.

#### 2.4.1 Socio-demographic characteristics

The socio-demographic characteristics included age, gender, marital status, educational level, occupation, and area of residence.

#### 2.4.2 Levenson self-report psychopathy scale

The LSRP is a 26-item scale that measures primary (affective and interpersonal) and secondary (behavioral) psychopathy (16). The LSRP has been validated in both community (16, 19, 20) and forensic settings (21). We used the 25-item LSRP scale, validated in a non-institutionalized population in Lebanon as a three-factor model rather than the original two-factor model (primary and secondary psychopathy) (17).

#### 2.4.3 TAPS-1 screening tool

Tobacco, Alcohol, Prescription medication, and other Substance use Part 1 (TAPS-1) is a four-item instrument assessing past-year use of tobacco, alcohol, illicit drugs, and non-medical prescription medications (22). Each item is coded “Yes” or “No.” As TAPS-1 is not available in Arabic, it was translated and backtranslated. An overall TAPS total score was then calculated by adding all four items, yielding a range of 0 (no substance use) to 4 (use of all four substances). Although it has not been validated in the Lebanese/Arabic context, TAPS-1 was employed for its brevity and clinical relevance. The results should be interpreted with caution given potential cultural and linguistic differences.

#### 2.4.4 Short version (20 items) of the urgency, premeditation (lack of), perseverance (lack of), sensation seeking, positive urgency, impulsive behavior scale

The short version of the Urgency, Premeditation (lack of), Perseverance (lack of), Sensation seeking, Positive urgency, Impulsive behavior (S-UPPS-P) scale is a 20-item self-report questionnaire designed to measure the following five facets of impulsivity: positive urgency, negative urgency, lack of premeditation, lack of perseverance, and sensation seeking. The English and Arabic versions, which have been validated in Lebanon, were used (23).

#### 2.4.5 Adverse childhood experiences

The Adverse Childhood Experiences (ACE) questionnaire is a 10-item self-report questionnaire that measures experiences of abuse, neglect, and household dysfunction. Each item was recoded to a dichotomous Yes/No variable. Higher total scores indicate greater exposure to adversity (24).

### 2.5 Statistical analysis

Descriptive analyses were first performed to summarize the sociodemographic characteristics of the participants. Categorical variables were presented as frequencies and percentages, while continuous variables were described using means and standard deviations (SD). Pearson’s chi-square tests and Student’s *t*-tests were used to examine bivariate associations between the demographic factors and each substance use category (tobacco, alcohol, illicit drugs, and prescription medication use).

We explored whether a summed TAPS total score could be analyzed ordinally by fitting an ordered logistic model and testing the proportional odds assumption through a likelihood-ratio test comparing the ordered model to a multinomial logistic model. The proportional odds assumption was not violated (the *χ*² likelihood-ratio ranged between 0.95 and 1.35, *p* > 0.9). Therefore, we proceeded with modeling the sum of substance use.

For each substance, a multivariable binary logistic regression model was fitted adjusting for the same set of covariates: age, sex (men *vs*. women), marital status, employment status, education level, the dose–response ACE score, primary and secondary psychopathy factors, and all five S-UPPS-P facets (i.e., negative urgency, positive urgency, lack of perseverance, lack of premeditation, and sensation seeking). The sample size for every model was added to the corresponding regression table. To guard against multicollinearity, variance inflation factors (VIFs) were calculated and values ≥4 considered as potentially problematic. Model calibration was assessed using the Hosmer–Lemeshow goodness-of-fit test and discrimination using the area under the receiver operating characteristic (AUC) curve. For the continuous psychopathy and impulsivity scales, standardized odds ratios per standard deviation (OR per SD) were calculated to aid interpretation. As multiple predictors were examined across several outcomes, the false discovery rate was controlled at 5% using the Benjamini–Hochberg procedure. Missingness across all variables was below 4%. Given this minimal proportion and the absence of systematic patterns, complete case analysis was used as imputation was not expected to meaningfully alter the estimates. Analyses were conducted in Stata 18.

## 3 Results

### 3.1 Sample characteristics

A total of 534 Lebanese participants were included. Of these, one-fifth comprised men, consistent with the typically higher female participation in online surveys, and more than half were single. Approximately 60% were employed, and 91% had completed university education. Detailed demographic information is presented in **Table 1**.

**Table 1:** Association between socio-demographic characteristics and substance use.

Variable	Total	Tobacco		Alcohol		Drugs		Prescription drugs	
		Yes	No	Yes	No	Yes	No	Yes	No
Age (years)	36.58 ± 11.94	37.01 ± 11.16	36.21 ± 12.59	35.22 ± 10.68	37.09 ± 12.36	33.87 ± 9.9	37.37 ± 12.37	36.37 ± 10.22	36.59 ± 12.15
*p*-value		0.43		0.11		**0.006**		0.94	
Sex									
Male	110 (20.87%)	57 (23.36%)	53 (18.73%)	33 (24.09%)	77 (19.85%)	31 (27.68%)	79 (19.36%)	16 (27.12%)	94 (20.09%)
Female	417 (79.13%)	187 (76.64%)	230 (81.27%)	104 (75.91%)	311 (80.15%)	81 (72.32%)	329 (80.64%)	43 (72.88%)	374 (79.91%)
*p*-value		0.19		0.29		0.06		0.21	
Marital status									
Single	274 (51.31%)	121 (48.59%)	153 (53.68%)	76 (54.29%)	197 (50.26%)	67 (58.26%)	203 (49.27%)	25 (41.67%)	249 (52.53%)
Married	230 (43.07%)	112 (44.98%)	118 (41.4%)	58 (41.43%)	172 (43.88%)	44 (38.26%)	183 (44.42%)	29 (48.33%)	201 (42.41%)
Separated	5 (0.94%)	2 (0.8%)	3 (1.05%)	2 (1.43%)	3 (0.77%)	1 (0.87%)	4 (0.97%)	0 (0)	5 (1.05%)
Divorced	23 (4.31%)	14 (5.62%)	9 (3.16%)	4 (2.86%)	18 (4.59%)	3 (2.61%)	20 (4.85%)	6 (10%)	17 (3.59%)
Widowed	2 (0.37%)	0 (0)	2 (0.7%)	0 (0)	2 (0.51%)	0 (0)	2 (0.49%)	0 (0)	2 (0.42%)
*p*-value		0.31		0.66		0.43		0.11	
Employment									
Unemployed	84 (15.73%)	47 (18.88%)	37 (12.98%)	32 (22.86%)	52 (13.27%)	22 (19.13%)	61 (14.81%)	11 (18.33%)	73 (15.4%)
Employed	323 (60.49%)	164 (65.86%)	159 (55.79%)	84 (60%)	237 (60.46%)	73 (63.48%)	245 (59.47%)	39 (65%)	284 (59.92%)
Student	87 (16.29%)	27 (10.84%)	60 (21.05%)	16 (11.43%)	71 (18.11%)	17 (14.78%)	69 (16.75%)	7 (11.67%)	80 (16.88%)
Homemaker	17 (3.18%)	4 (1.61%)	13 (4.56%)	3 (2.14%)	14 (3.57%)	2 (1.74%)	15 (3.64%)	2 (3.33%)	15 (3.16%)
Retired	23 (4.31%)	7 (2.81%)	16 (5.61%)	5 (3.57%)	18 (4.59%)	1 (0.87%)	22 (5.34%)	1 (1.67%)	22 (4.64%)
*p*-value		**0.001**		**0.04**		0.16		0.64	
Education									
Less than high school	5 (0.94%)	3 (1.2%)	2 (0.7%)	1 (0.71%)	4 (1.02%)	0 (0)	5 (1.21%)	1 (1.67%)	4 (0.84%)
High school	28 (5.24%)	10 (4.02%)	18 (6.32%)	2 (1.43%)	26 (6.63%)	1 (0.87%)	26 (6.31%)	4 (6.67%)	24 (5.06%)
Technical school	14 (2.62%)	7 (2.81%)	7 (2.46%)	2 (1.43%)	12 (3.06%)	2 (1.74%)	12 (2.91%)	3 (5%)	11 (2.32%)
University	487 (91.2%)	229 (91.97%)	258 (90.53%)	135 (96.43%)	350 (89.29%)	112 (97.39%)	369 (89.56%)	52 (86.67%)	435 (91.77%)
*p*-value		0.62		0.07		0.05		0.52	

Bold: statistically significant results

### 3.2 Correlation between socio-demographic characteristics and substance use

We examined the associations between participant characteristics and each substance use category (tobacco, alcohol, illicit drugs, and prescription drug misuse). Age was significantly associated with illicit drug use (*p* = 0.006), with younger participants (mean = 33.87 ± 9.9) more likely to report drug use than older participants (37.37 ± 12.37). Employment status showed significant relationships with tobacco (*p* = 0.001) and alcohol (*p* = 0.04), with unemployed participants reporting higher use of both tobacco (18.88% *vs*. 12.98% among non-users) and alcohol (22.86% *vs*. 13.27%) (**Table 1**).

### 3.3 LSRP and TAPS analysis

Initial analyses of the psychopathy traits revealed distinct patterns of association with substance use. Higher callousness scores showed consistent relationships across multiple substances, demonstrating significant associations with tobacco (*p* = 0.010), alcohol (*p* = 0.002), and illicit drug use (*p* = 0.008). In contrast, both the egocentric and antisocial subscales showed specificity in their relationships, being predominantly linked to prescription drug misuse (*p* = 0.002 and *p* < 0.001, respectively) (**Table 2**).

**Table 2:** Bivariate analysis for the association of TAPS (tobacco, alcohol, prescription medication, and other substance use screener) and psychopathy.

mean±S.D	Callous	Egocentric	Antisocial	Primary	Total
*t*-test					
TAPS total score					
Yes	9.87 ± 2.75	21.42 ± 5.08	18.71 ± 3.39	29.04 ± 6.44	50 ± 8.32
No	9.37 ± 2.32	20.34 ± 4.99	17.52 ± 3.41	27.57 ± 5.94	47.23 ± 8.54
*p*-value	**0.03**	**0.02**	**<0.001**	**0.008**	**<0.001**
Tobacco					
Yes	9.94 ± 2.75	21.61 ± 5.16	18.88 ± 3.38	29.28 ± 6.49	50.43 ± 8.35
No	9.38 ± 2.37	20.33 ± 4.9	17.57 ± 3.38	27.56 ± 5.92	47.27 ± 8.37
*p*-value	**0.01**	**0.003**	**<0.001**	**0.002**	**<0.001**
Alcohol					
Yes	10.21 ± 2.48	21.35 ± 4.76	18.26 ± 3.26	29.34 ± 5.88	49.83 ± 7.32
No	9.43 ± 2.57	20.76 ± 5.17	18.15 ± 3.5	27.99 ± 6.36	48.34 ± 8.87
*p*-value	**0.002**	0.23	0.74	**0.03**	0.08
Drugs					
Yes	10.22 ± 2.54	21.09 ± 4.79	18.21 ± 3.34	29.09 ± 5.97	49.51 ± 7.35
No	9.49 ± 2.56	20.93 ± 5.14	18.18 ± 3.49	28.22 ± 6.32	48.6 ± 8.81
*p*-value	**0.008**	0.77	0.94	0.19	0.31
Prescription drugs					
Yes	9.95 ± 3.13	22.4 ± 5.82	20.72 ± 3.21	29.93 ± 7.28	53.07 ± 9.42
No	9.59 ± 2.48	20.74 ± 4.93	17.86 ± 3.33	28.16 ± 6.08	48.19 ± 8.23
*p*-value	0.32	**0.02**	**<0.001**	**0.04**	**<0.001**
ANOVA					
TAPS total score					
0	9.37 ± 2.32	20.23 ± 4.99	17.52 ± 3.41	27.57 ± 5.94	47.23 ± 8.54
1	9.47 ± 2.65	21.06 ± 4.81	18.73 ± 3.25	28.33 ± 6.09	49.27 ± 8.15
2	10.02 ± 3.24	22.05 ± 6.14	18.52 ± 3.91	29.67 ± 7.97	50.59 ± 10.25
3	10.35 ± 2.43	21.63 ± 4.63	18.25 ± 3.17	29.67 ± 5.84	50.22 ± 7.01
4	10.47 ± 2.34	21.26 ± 4.99	20.84 ± 2.69	29.68 ± 4.93	52.58 ± 6.48
*p*-value	**0.01**	0.13	0.24	**0.02**	**0.02**
Univariate ordinal regression					
TAPS total score					
Unadjusted OR	1.09	1.04	1.09	1.04	1.04
*p*-value	**0.002**	**0.01**	**<0.001**	**0.002**	**<0.001**
Spearman					
TAPS total score					
Rho	0.12	0.11	0.15	0.04	0.18
*p*-value	**0.006**	**0.009**	**<0.001**	0.32	**<0.001**

Bold: statistically significant results

When examining the relationship between cumulative substance use and psychopathy traits through the TAPS total score categories, a progressive pattern was observed. As substance use increased from none to multiple substances, there were significant increases in the callousness (9.37 ± 2.32 *vs*. 10.47 ± 2.34, *p* = 0.01) and total psychopathy scores (47.23 ± 8.54 *vs*. 52.58 ± 6.48, *p* = 0.02). However, this dose–response relationship did not extend to the egocentric (*p* = 0.13) or antisocial (*p* = 0.24) factors, suggesting that these characteristics may function independently of the number of substances used (**Table 2**).

After controlling for the socio-demographic factors and impulsivity, the regression analyses revealed more nuanced relationships between specific psychopathy dimensions and substance use patterns. The antisocial factor emerged as particularly strongly associated with overall substance use (OR per SD = 1.27, 95% CI = 1.04–1.55, *p* = 0.02) and tobacco use specifically (OR per SD = 1.51, 95% CI = 1.18–1.92, *p* = 0.001). Most notably, individuals with elevated antisocial scores showed the strongest likelihood of prescription drug misuse (OR per SD = 2.33, 95% CI = 1.57–3.45, *p* < 0.001) (**Table 3**).

**Table 3:** Binary and ordinal logistic regression models for the association between TAPS (tobacco, alcohol, prescription medication, and other substance use screener) and psychopathy traits, controlling for other confounding variables.

Variable	TAPS total score	Tobacco	Alcohol	Drugs	Prescription drugs
	Ordinal (*N* = 527)	Logistic (*N* = 525)	Logistic (*N* = 523)	Logistic (*N* = 513)	Logistic (*N* = 520)
Callous					
OR (standardized OR)	1.18	1.13	1.42	1.37	0.81
*p*-value	0.07	0.26	**0.003**	**0.01**	0.24
95% CI	0.98–1.42	0.91–1.40	1.13–1.79	1.07–1.75	0.57–1.14
Egocentric					
OR (standardized OR)	1.03	1.1	0.94	0.91	1.09
*p*-value	0.78	0.42	0.69	0.54	0.61
95% CI	0.83–1.27	0.87–1.39	0.73–1.23	0.69–1.20	0.77–1.56
Antisocial					
OR (standardized OR)	1.27	1.5	0.88	1.01	2.33
*p*-value	**0.02**	**0.001**	0.33	0.95	**<0.001**
95% CI	1.04–1.55	1.18–1.92	0.67–1.14	0.76–1.33	1.58–3.45
Age (years)					
OR	0.99	1.01	0.98	0.97	0.99
*p*-value	0.34	0.64	0.08	0.02	0.93
95% CI	0.97–1.01	0.98–1.03	0.95–1.01	0.94–0.99	0.96–1.04
Sex					
OR	1.19	1.12	0.95	1.29	1.52
*p*-value	0.39	0.63	0.84	0.34	0.26
95% CI	0.79–1.82	0.69–1.18	0.56–1.61	0.76–2.21	0.73–3.16
Marital status					
Married					
OR	1.49	1.46	1.11	1.12	2.97
*p*-value	0.06	0.11	0.7	0.69	0.008
95% CI	0.98–2.27	0.91–2.33	0.65–1.87	0.54–1.96	1.32–6.66
Separated					
OR	0.94	0.92	1.38	0.77	1
*p*-value	0.95	0.93	0.75	0.82	–
95% CI	0.15–5.76	0.14–6.09	0.19–9.99	0.07–7.59	–
Divorced					
OR	1.69	1.93	0.78	0.8	4.24
*p*-value	0.21	0.19	0.69	0.75	0.03
95% CI	0.74–3.89	0.72–5.14	0.22–2.73	0.21–3.06	1.19–15.15
Widowed					
OR	1	1	1	1	1
*p*-value	–	–	–	–	–
95% CI	–	–	–	–	–
Employment Employed					
OR	0.85	0.95	0.54	0.81	1.35
*p*-value	0.5	0.86	0.03	0.49	0.46
95% CI	0.54–1.36	0.56–1.61	0.31–0.94	0.45–1.47	0.6–3.04
Student					
OR	0.43	0.46	0.24	0.44	0.95
*p*-value	0.01	0.04	0.001	0.06	0.94
95% CI	0.22–0.82	0.22–0.96	0.11–0.55	0.19–1.02	0.28–3.19
Homemaker					
OR	0.39	0.2	0.38	0.49	1.17
*p*-value	0.09	0.02	0.2	0.41	0.86
95% CI	0.23–1.16	0.05–0.76	0.09–1.67	0.09–2.59	0.19–6.92
Retired					
OR	0.47	0.29	0.74	0.22	0.26
*p*-value	0.1	0.03	0.63	0.17	0.24
95% CI	0.18–1.17	0.1–0.89	0.22–2.48	0.03–1.87	0.03–2.49
Education level High school					
OR	0.97	0.79	0.31	0.09	4.48
*p*-value	0.98	0.83	0.39	0.03	0.32
95% CI	0.17–5.45	0.09–6.42	0.02–4.68	0.01–0.78	0.24–83.84
Technical school					
OR	2.52	2.1	0.89	0.66	8.34
*p*-value	0.32	0.51	0.94	0.61	0.17
95% CI	0.41–15.38	0.23–10.08	0.06–13.96	0.13–3.28	0.41–171.67
University					
OR	2.08	1.39	1.95	1	2.43
*p*-value	0.36	0.73	0.56	–	0.52
95% CI	0.44–9.91	0.21–9.37	0.21–18.6	–	0.17–35.66
ACE total score					
OR	1.09	1.23	1.07	1.04	0.9
*p*-value	0.14	**0.005**	0.43	0.63	0.36
95% CI	0.97–1.25	1.07–1.43	0.91–1.25	0.88–1.23	0.72–1.13
Negative urgency					
OR	1.02	0.99	1.01	1.01	1.19
*p*-value	0.66	0.77	0.83	0.98	**0.02**
95% CI	0.94–1.01	0.89–1.08	0.91–1.12	0.89–1.12	1.02–1.37
Positive urgency					
OR	1.01	1.01	1.08	0.99	0.87
*p*-value	0.85	0.95	0.19	0.83	0.08
95% CI	0.92–1.1	0.91–1.11	0.96–1.21	0.88–1.11	0.74–1.02
Lack of perseverance					
OR	0.93	0.87	0.99	0.98	1.07
*p*-value	0.16	**0.02**	0.98	0.77	0.45
95% CI	0.85–1.03	0.78–0.98	0.88–1.13	0.87–1.11	0.9–1.27
Lack of premeditation					
OR	1.01	1.06	0.89	0.92	1.11
*p*-value	0.99	0.35	0.07	0.23	0.27
95% CI	0.91–1.11	0.94–1.19	0.78–1.01	0.81–1.05	0.93–1.32
Sensation seeking					
OR	1.11	1.11	1.115	1.11	1.13
*p*-value	**0.001**	**0.006**	**0.001**	**0.02**	**0.04**
95% CI	1.04–1.19	1.03–1.19	1.06–1.25	1.02–1.2	1.01–1.27

Bold: statistically significant results

Callousness maintained its significant relationships in the adjusted analyses, with higher scores predicting both alcohol use (OR per SD = 1.42, 95% CI = 1.13–1.79, *p* = 0.003) and illicit drug use (OR per SD = 1.37, 95% CI = 1.07–1.75, *p* = 0.01). These adjusted findings reinforce the distinct patterns observed in the initial analyses, confirming that specific psychopathy dimensions, particularly the callous and antisocial factors, show consistent and robust associations with different forms of substance use. The model discrimination was acceptable, with AUC values of approximately 0.71 for tobacco, 0.69 for alcohol, 0.69 for illicit drugs, and 0.78 for prescription medications. The results of the Hosmer–Lemeshow tests suggested adequate calibration for all models. After adjustment for the false discovery rate with the Benjamini–Hochberg procedure, the association between the antisocial factor and the TAPS total score lost its statistical significance (**Table 3**).

## 4 Discussion

This study provides the first evidence of how psychopathy traits relate to substance use patterns in Lebanon, revealing relationships that both align with and diverge from Western findings. Within Lebanon’s unique cultural and economic contexts, distinct patterns were found: callousness was associated with alcohol and illicit drug use, while the antisocial factor was associated with tobacco and prescription drug misuse. These relationships must be understood within the context of Lebanon’s current challenges, including economic instability and healthcare system strain.

These patterns show important demographic variations. It was found that younger participants were more likely to use substances than older participants. This is consistent with global trends, where substance use disorders (e.g., alcohol, tobacco, cannabis, or opioid) generally decrease with age (6). The majority of our participants were over 30 years old. In a study done in Lebanon by Karam et al. (25), substance use was strongly associated with young age (18–34 years; OR = 14.5). Another 2019 study reported a 12-month drug abuse prevalence of less than 0.1% in Lebanon, with higher rates among individuals aged 18–29 years compared with those 60 years and older (26). Furthermore, our research found no statistical significance between men and women regarding drug use. This contrasts with regional trends, where a study among Moroccan adolescents (27) and another done in the United Arab Emirates (28) reported higher substance use among men. Due to the predominance of women in our sample (79.13%), caution is warranted when generalizing these findings to the general Lebanese population. Increasing stressors exacerbated by the economic instability faced by the Lebanese population could have also led both genders to engage in substance use at similar rates. Higher rates of alcohol and tobacco use were found in unemployed individuals, similar to prior research conducted in the region (29). Beyond demographics, the relationship between specific psychopathy traits and substance use revealed complex patterns that reflect both global trends and local contexts.

Early evidence suggests that substance use is highly linked to the antisocial and lifestyle facets of psychopathy, known as factor 2 of the PCL-R (30, 31). Nonetheless, more recent studies have examined the association between substance use and factor 1 (interpersonal and affective facets) of psychopathy. Denomme et al. (32) concluded that such traits may predispose individuals to a heightened neural response to drug-related stimuli, potentially increasing the risk of substance use disorders.

Callousness is an affective trait of psychopathy that is characterized by the lack of remorse and empathy. While our results showed that callousness increases the risk of illicit drug and alcohol use, the findings in the literature are mixed. For instance, Walsh et al. (33) reported an association between callous interpersonal style and cocaine use. Moreover, the association between chronic marijuana use and callous traits was highlighted by Pardini et al. (34). Similarly, Coid et al. (35) discovered a positive association between affective traits and lifetime use of cocaine, sedatives, and solvents. Individuals with callous personality traits are often manipulative, unemotional, and driven by personal gain, which aligns with risk-taking behaviors, including illicit drug use (34). A study that utilized the LSRP in a sample of college students described a relationship between primary psychopathy and alcohol use and alcohol problems (36). Moreover, a 2022 study that explored the relationship between different personality traits and alcohol use disorder highlighted the association between alcohol use disorder and callousness (37). Psychopaths with callous traits exhibit reduced harm avoidance, limiting their use of protective behavioral strategies and increasing the risk of alcohol use (36). On the other hand, Walsh et al. (33) discovered a negative association between the affective facet and symptoms of cannabis dependence. Moreover, in their study on two independent samples of inmates, Brieman et al. (38) reported that affective traits were negatively related to the severity of illicit substance (i.e., cannabis, opioids, and cocaine) and alcohol dependence. This discrepancy in the findings of different studies could be attributed to multiple factors. One factor would be the use of different tools and/or frameworks to measure psychopathy, such as the PCL-R (32, 33, 35, 38) and LSRP (36). Another contributing factor could be the differences in the study populations, including socioeconomic or educational status and community *versus* forensic samples. Interestingly, Kramer et al. used the LSRP in a sample of college students, similar to our use of LSRP in our sample of non-institutionalized individuals (91.2% university level), which showed similar findings of an increase in alcohol use linked to primary psychopathy/callousness. However, the callous factor was not associated with prescription drug misuse, in contrast with the findings from male prison inmates where affective traits correlated with sedative use (35). In the past few years, Lebanon has passed through multiple crises, including the economic crisis, the COVID-19 pandemic, and the Beirut Port explosion. These crises have led to medication shortages and increased costs, making prescription drugs less accessible (39). Individuals with callousness may be deterred by these barriers, as their impulsivity and their need for immediate gratification (40) lead them to prefer illicit substances that are more readily available in Lebanon, such as Captagon or cannabis (41). Furthermore, cultural attitudes in Lebanon might have influenced substance use patterns. While the misuse of prescription drugs might lead to social stigmatization and legal consequences, the use of tobacco and alcohol is more socially tolerated (42).

In this study, the antisocial factor was unrelated to alcohol use, diverging from prior findings (33, 35). However, it was linked to prescription drug misuse, supporting an association with opioids, sedatives, and stimulants (35). In Lebanon, such individuals might exploit gaps in the healthcare system to access prescription drugs without proper medical oversight. In a study on university students in Lebanon, approximately one in five students admitted to non-medical use of prescription medication. It was found that the second most reported source for sleep and pain medications is from a pharmacist without a prescription, second only to use of parental medication. Of the students with a prescription medication, 20% diverted their medication. Moreover, up to 63% reported that it would be easy to obtain prescription drugs (43). We also found a link between the antisocial factor and higher tobacco use, similar to a study among incarcerated offenders (44). This association may arise because individuals with a secondary factor, characterized by impulsivity, use nicotine to manage negative emotions. The effects of nicotine, such as relaxation, anxiety reduction, and stress relief, are used as a coping mechanism, potentially leading to problematic use patterns (45, 46). In line with this, deficits in cognitive responsibility, moral reasoning, and intellectual development have also been shown to influence antisocial and substance-related behaviors, extending the interpretation of these traits beyond purely behavioral correlates (47).

These findings demand a shift from generic approaches to targeted strategies within Lebanon’s strained healthcare system. Clinically, the integration of personality trait assessments into substance use screening would allow overburdened clinicians to allocate resources more effectively. For policymakers, this could involve investing in public health campaigns that address specific risk traits and training frontline workers to recognize such traits. These steps could facilitate prompt interventions and create a more efficient, culturally coherent response to the nation’s substance use crisis.

### 4.1 Strengths and limitations

Our study has several strengths. Firstly, it examined the relationship between psychopathic traits and substance use in a Lebanese sample, contributing to a growing but limited research on personality disorders and substance use. By focusing on cultural and contextual factors, our study provides insights not previously captured in Western studies. Secondly, our study had a large sample size. Thirdly, we used the validated Lebanese version of the LSRP scale (17), which ensured reliable and accurate measurement of psychopathic traits.

Despite the importance of our results, this study has some limitations. The first limitation is the reliance on self-report and convenience recruitment through social media, which produced a predominantly female and highly educated sample, which may have introduced selection bias (48) and limited the generalizability of the results. Secondly, the TAPS scale is not a validated tool in the Arabic socio-cultural context. Thirdly, the TAPS is merely a screening tool, and it does not differentiate between occasional substance use and substance use. It is possible that we would have found stronger associations between psychopathy and substance use among individuals with more severe use disorders. Fourthly, given the cross-sectional design of the study, causal relationships cannot be established, and it is possible that the observed associations reflect reverse causality rather than a directional effect. Finally, the survey was conducted amid political and economic turmoil and post-COVID-19, which may have influenced the responses.

## 5 Conclusion

In conclusion, this study provides the first examination of the association between psychopathy and substance use in Lebanon and the broader Arab world. The findings revealed distinct patterns within Lebanon’s cultural context and indicated different substance use patterns, suggesting the need for culturally tailored intervention approaches. Given Lebanon’s current economic challenges and healthcare system strain, these insights could help target limited resources more effectively through early identification and intervention. Future research should extend these findings to forensic populations and develop or validate culturally appropriate screening tools, ultimately contributing to a better understanding of how personality disorders and substance use manifest in Arab cultures.

## Data Availability

The raw data supporting the conclusions of this article will be made available by the authors, without undue reservation.
